# Supplementary figures and images for: Metabolic profiling of Mytilus coruscus mantle in response of shell repairing under acute acidification
Source: PLoS One. 2023 Oct 27;18(10):e0293565. doi: 10.1371/journal.pone.0293565 (PMC10610157; doi:10.1371/journal.pone.0293565)

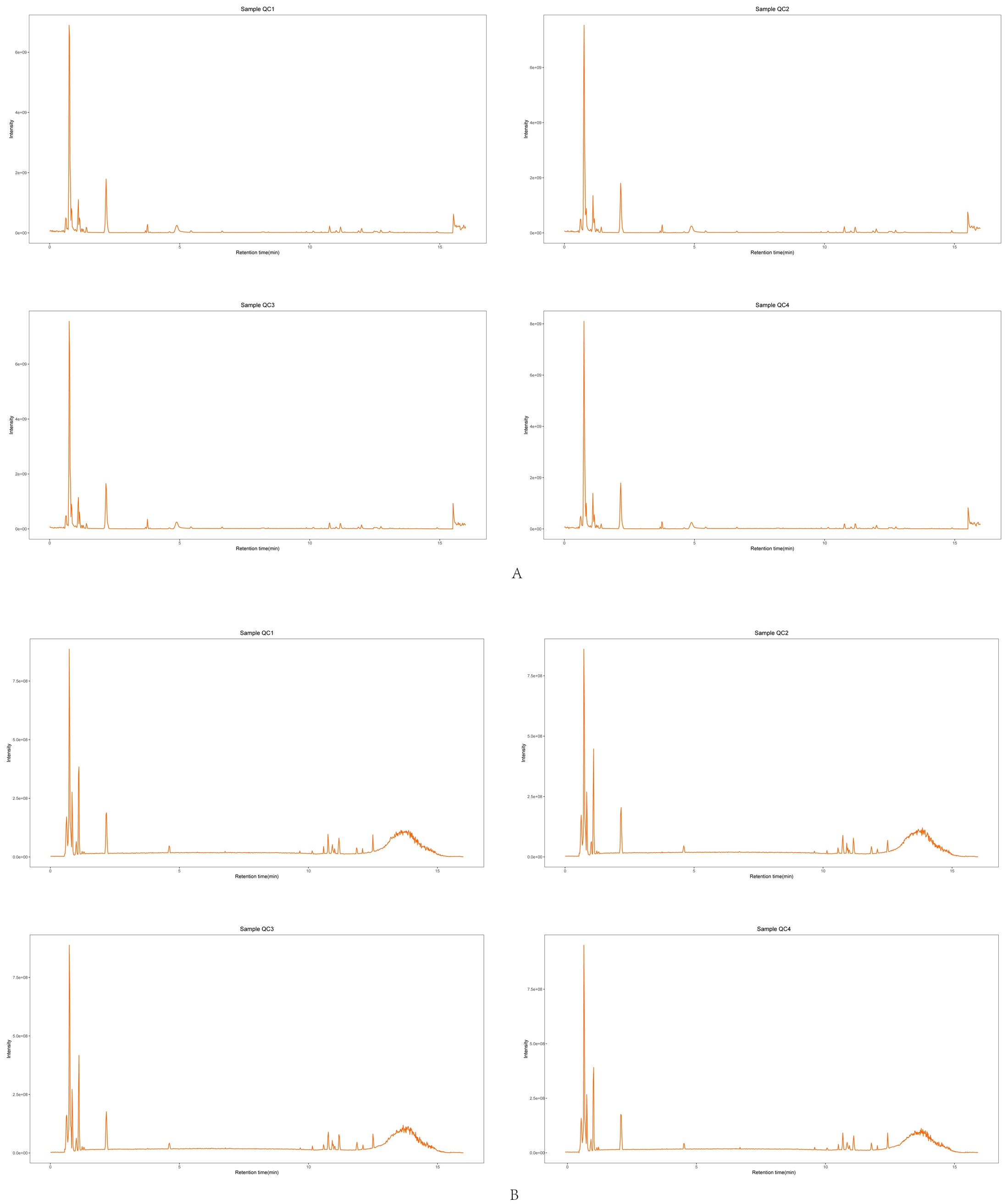

Supplement: S1 Fig — A ~ D: Ion chromatograms of QC samples under positive model for QC1 ~QC4, respectively; E ~ H: Ion chromatograms of QC samples under negative model for QC1 ~QC4, respectively. (TIF) [file pone.0293565.s001.tif]

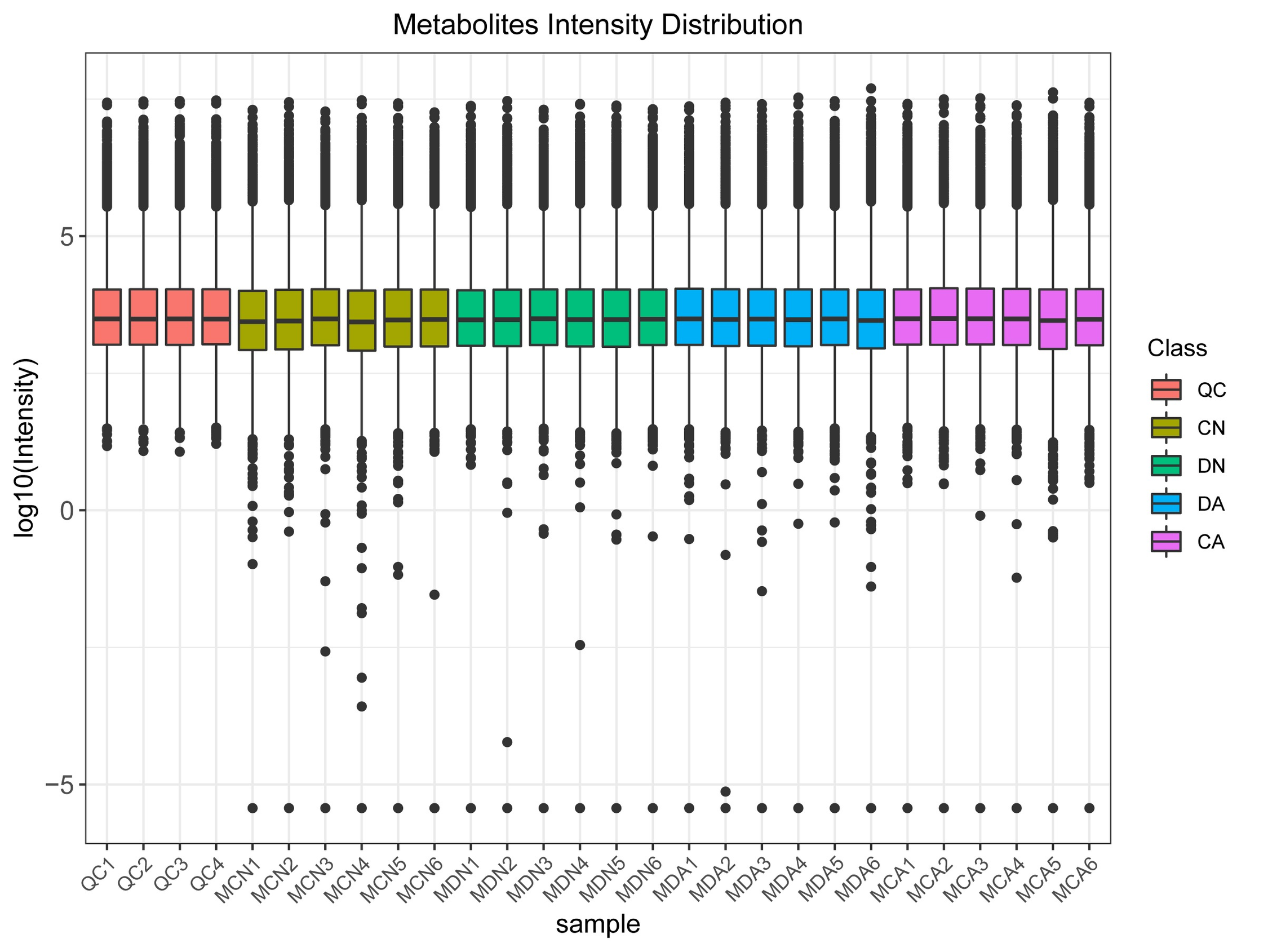

Supplement: S2 Fig — CN, the mussel with complete shell and fed in normal sea water (pH 8.1); DN, the mussel with drilled shell and fed in normal sea water (pH 8.1); CA, the mussel with complete shell and fed in acidified sea water (pH 7.4) with exposure time of 48 h; DA, the mussel with drilled shell and fed in acidified sea water (pH 7.4) with exposure time of 48 h. (TIF) [file pone.0293565.s002.tif]

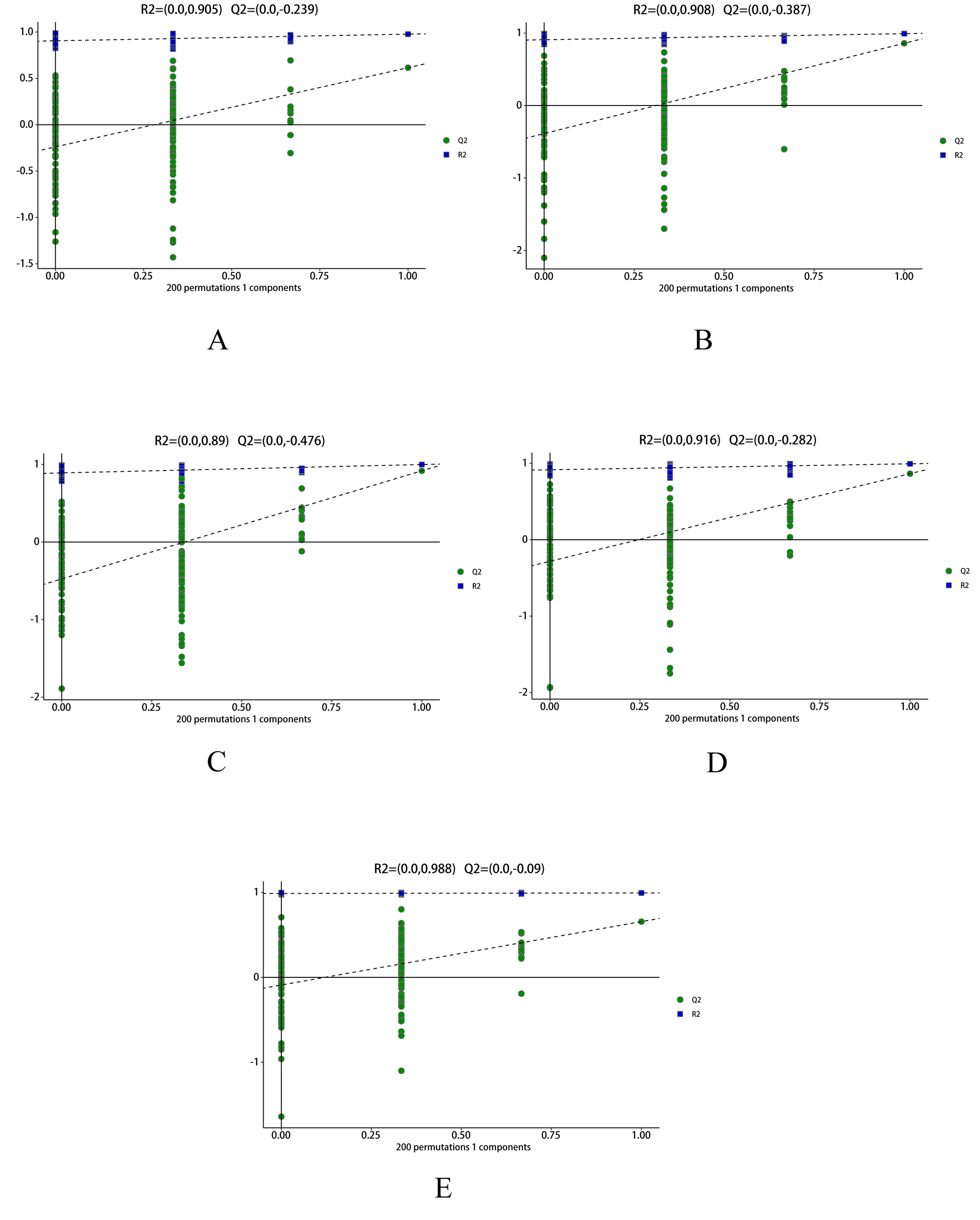

Supplement: S3 Fig — CN, the mussel with complete shell and fed in normal sea water (pH 8.1); DN, the mussel with drilled shell and fed in normal sea water (pH 8.1); CA, the mussel with complete shell and fed in acidified sea water (pH 7.4) with exposure time of 48 h; DA, the mussel with drilled shell and fed in acidified sea water (pH 7.4) with exposure time of 48 h. (A)DN vs CN; (B)DA vs CN; (C)CA vs CN; (D)DA vs DN; (E)CA vs DA. (TIF) [file pone.0293565.s003.tif]

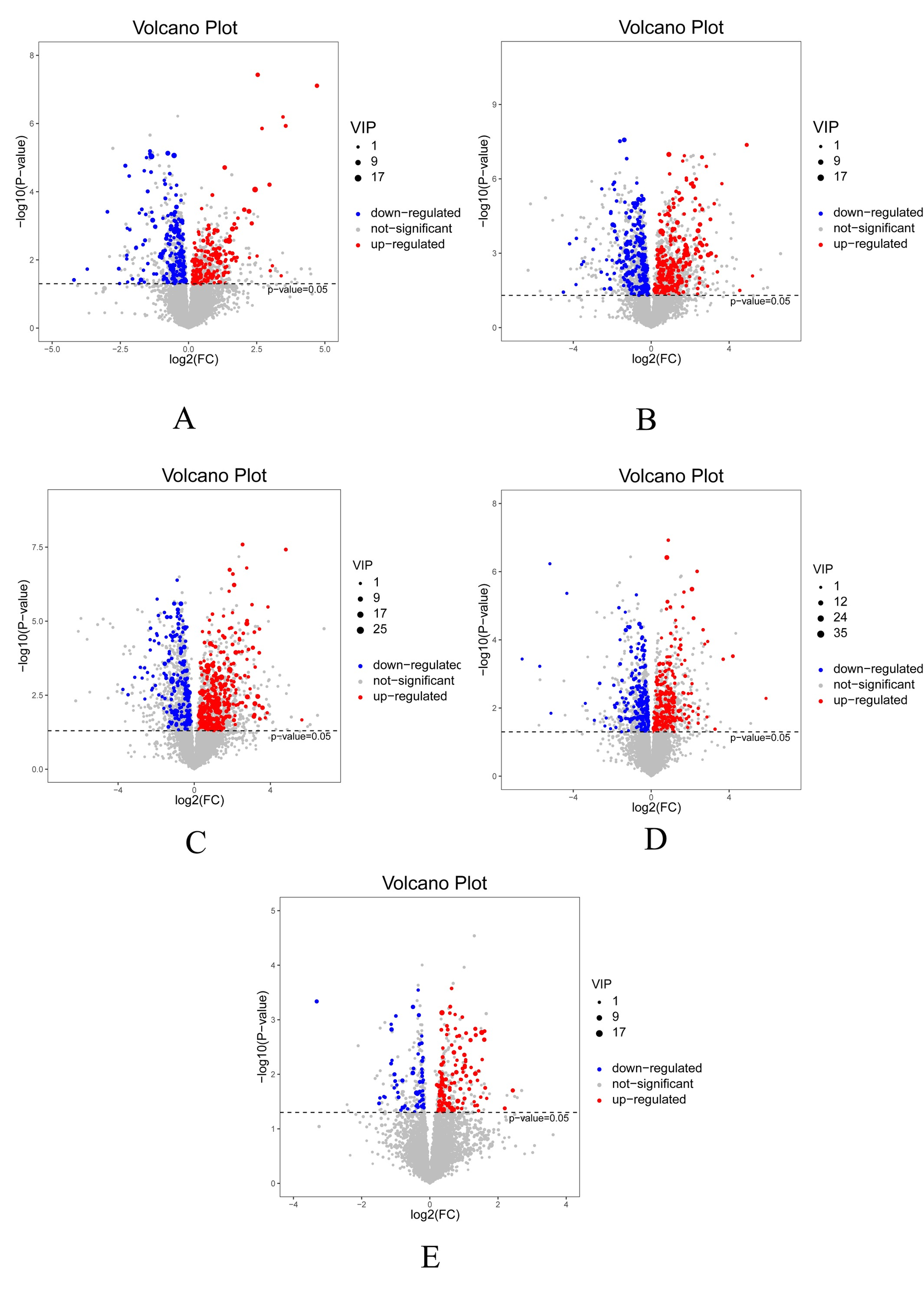

Supplement: S4 Fig — CN, the mussel with complete shell and fed in normal sea water (pH 8.1); DN, the mussel with drilled shell and fed in normal sea water (pH 8.1); CA, the mussel with complete shell and fed in acidified sea water (pH 7.4) with exposure time of 48 h; DA, the mussel with drilled shell and fed in acidified sea water (pH 7.4) with exposure time of 48 h. (A)DN vs CN; (B)DA vs CN; (C)CA vs CN; (D)DA vs DN; (E)CA vs DA. (TIF) [file pone.0293565.s004.tif]

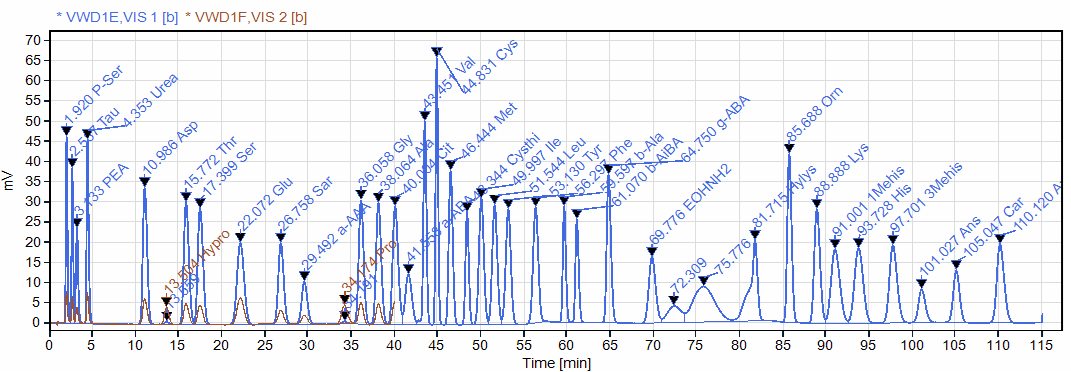

Supplement: S5 Fig — (TIF) [file pone.0293565.s005.tif]

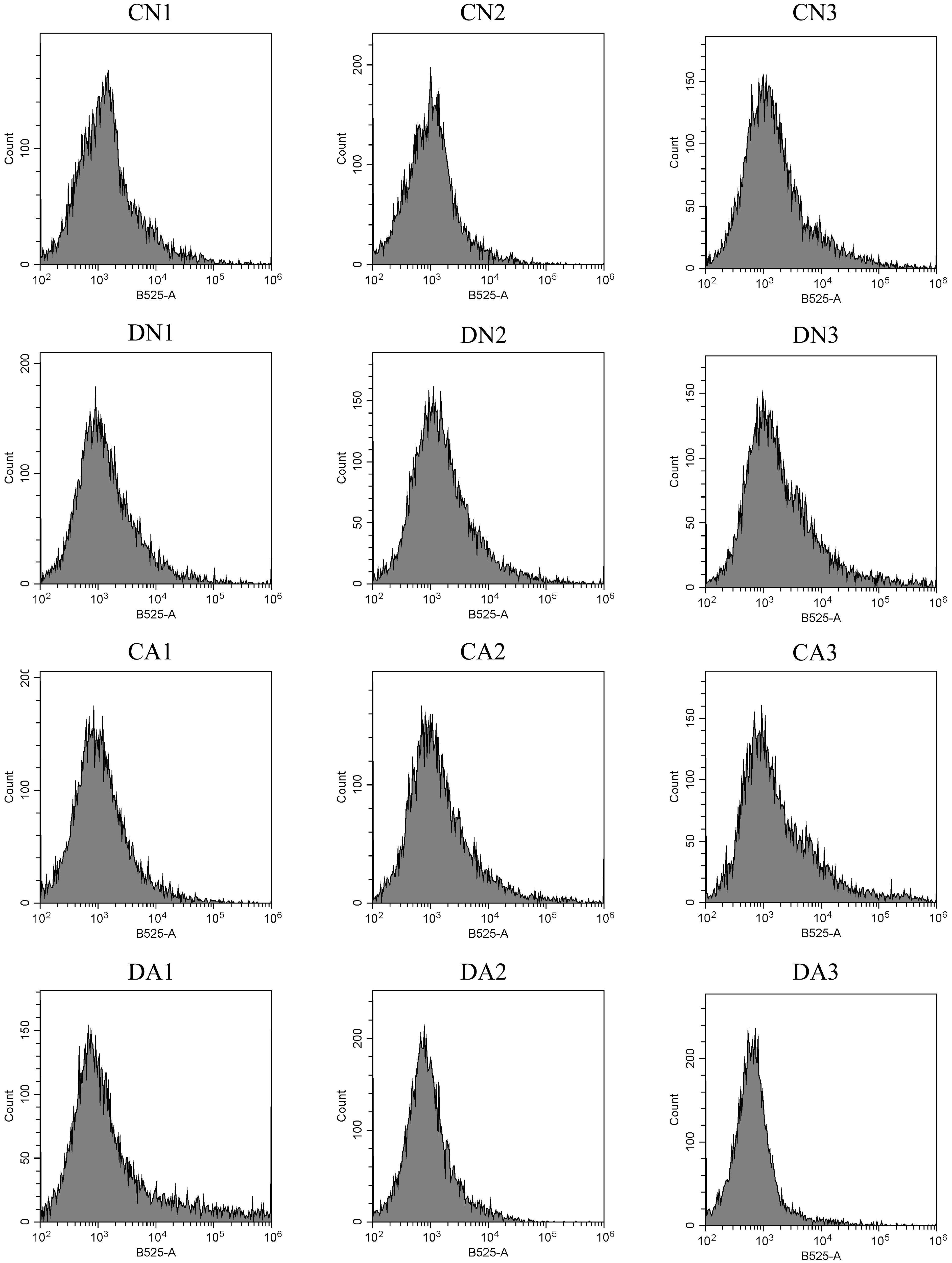

Supplement: S6 Fig — CN, the mussel with complete shell and fed in normal sea water (pH 8.1); DN, the mussel with drilled shell and fed in normal sea water (pH 8.1); CA, the mussel with complete shell and fed in acidified sea water (pH 7.4) with exposure time of 48 h; DA, the mussel with drilled shell and fed in acidified sea water (pH 7.4) with exposure time of 48 h. (TIF) [file pone.0293565.s006.tif]

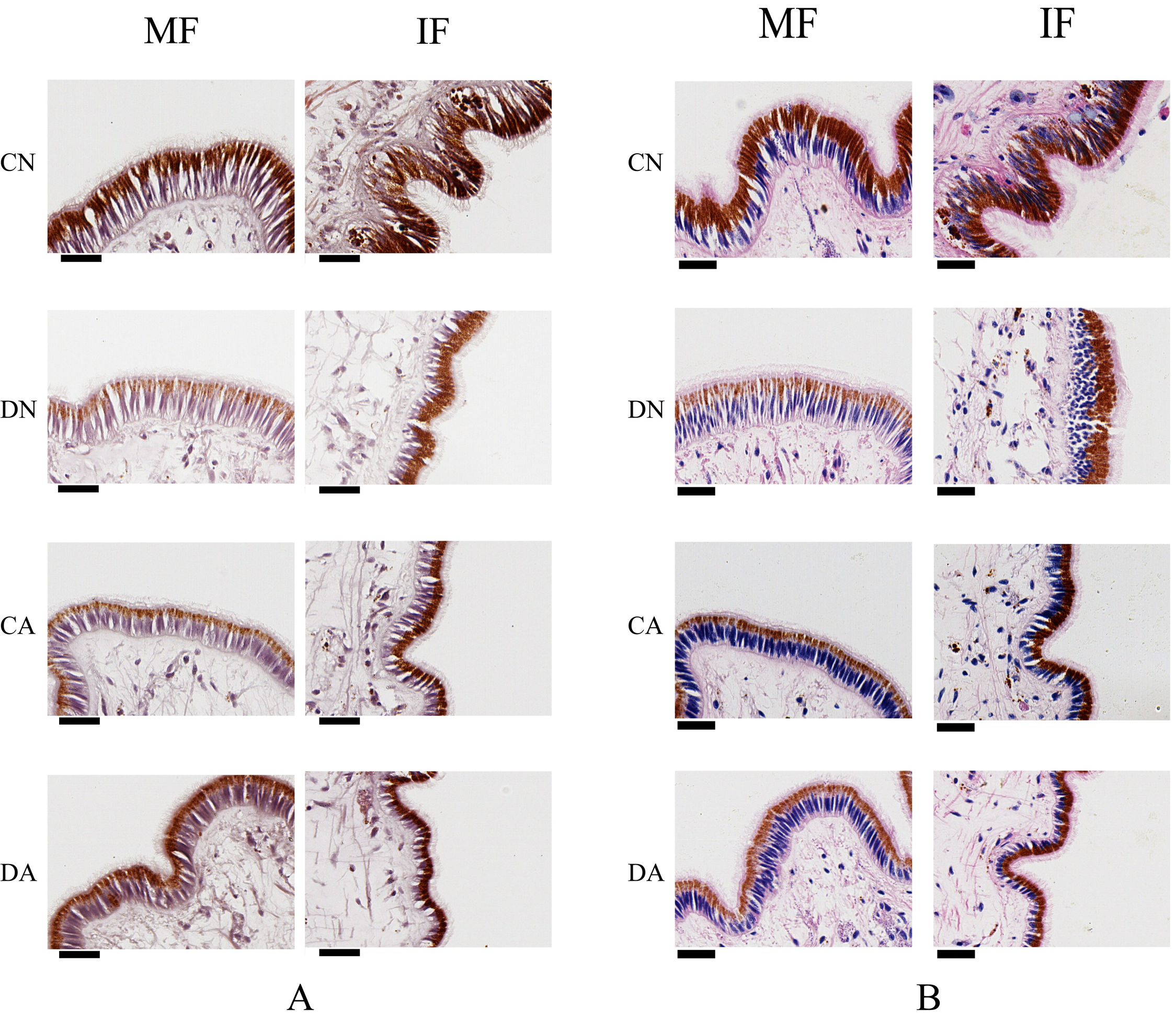

Supplement: S7 Fig — A: the mantle edge was cut at 4μm and stained with ARS. B: the mantle edge was cut at 4μm and stained with Von Kossa. CN, the mussel with complete shell and fed in normal sea water (pH 8.1); DN, the mussel with drilled shell and fed in normal sea water (pH 8.1); CA, the mussel with complete shell and fed in acidified sea water (pH 7.4) with exposure time of 48 h; DA, the mussel with drilled shell and fed in acidified sea water (pH 7.4) with exposure time of 48 h. (TIF) [file pone.0293565.s007.tif]
