# Supplementary material for: Metabolic profiling of Mytilus coruscus mantle in response of shell repairing under acute acidification
Source: PLoS One. 2023 Oct 27;18(10):e0293565. doi: 10.1371/journal.pone.0293565 (PMC10610157; doi:10.1371/journal.pone.0293565)
Supplement: S1 Table — CN, the mussel with complete shell and fed in normal sea water (pH 8.1); DN, the mussel with drilled shell and fed in normal sea water (pH 8.1); CA, the mussel with complete shell and fed in acidified sea water (pH 7.4) with exposure time of 48 h; DA, the mussel with drilled shell and fed in acidified sea water (pH 7.4) with exposure time of 48 h. (DOCX) [file pone.0293565.s008.docx]

| **Group** | **Type** | **R2X(cum)** | **R2Y(cum)** | **Q2(cum)** | **R2** | **Q2** |
| --- | --- | --- | --- | --- | --- | --- |
| CN vs CS | OPLS | 0.691 | 0.977 | 0.615 | 0.905 | -0.239 |
| AS vs CN | OPLS | 0.747 | 0.992 | 0.859 | 0.908 | -0.387 |
| AN vs CN | OPLS | 0.722 | 0.998 | 0.917 | 0.89 | -0.476 |
| AS vs CS | OPLS | 0.711 | 0.994 | 0.865 | 0.916 | -0.282 |
| AN vs AS | OPLS | 0.779 | 0.994 | 0.656 | 0.988 | -0.09 |
