# Supplementary material for: Metabolic profiling of Mytilus coruscus mantle in response of shell repairing under acute acidification
Source: PLoS One. 2023 Oct 27;18(10):e0293565. doi: 10.1371/journal.pone.0293565 (PMC10610157; doi:10.1371/journal.pone.0293565)
Supplement: Attachment — Submitted filename: Responses to reviwers.docx [file pone.0293565.s009.docx]

**Responses to reviewers**

We express our gratitude to the reviewers and editors for their valuable comments. We are glad that the reviewers give us the opportunity to upload a revised version of our manuscripts. Below there are our responds to the reviewer´s comments.

**Review Comments to the Author**

Reviewer #1: The manuscript entitled “Metabolic profiling of Mytilus coruscus mantle in response of shell repairing under acute acidification” presents reasonable experimental design and data analysis process, and reports results of the physiological impact of shell damage and ocean acidification conditions on Mytilus coruscus. Overall, the authors' findings provide some interesting data for explaining the possible molecular responses of the mussel mantle to the low pH and shell damage, and thusly make it worthy of publication. Some specific comments were provided below. The authors are welcome to follow my suggestions for improving the manuscript.

**Introduction:**

Introduction is generally elucidative. The cited references are exhaustive and updated, and provide an adequate background regarding the topic of the research.

In a picky way, the following recently published article or book might be appropriate citations to further strengthen the background of this study.

https://doi.org/10.1016/j.aquatox.2020.105740

https://doi.org/10.1016/C2019-0-04274-4

**Response**: thanks for reviewer’s comments. We checked the references and one of them was added in Introduction as reference 21.

**Materials and Methods:**

The experimental design needs to be more detailed.

- How was the experimental system calibrated to ensure that the reported pH values (8.1 and 7.4) were precise?

- Additionally, could the authors please provide some type of environmental/ecological justification for the low pH value used?

- The authors stated that there are four groups of mussels used in this study. Was there pH treatment-level replication?

- were drilled/undrilled mussels that were reared in low pH all in the same low pH tank? - How many individuals for each group, and in each tank?

**Response**: thanks for reviewer’s suggestions. We rewrote the section and the detail information was added accordingly.

- In this study, the pH value was monitored using a pH meter, and adjusted using a CO2 pump to ensure the precise of pH value.

- The two pH levels used in this study were selected based on the average pH value at the local mussel farm and the estuarine habitat where *M. coruscus* lives in a highly ﬂuctuating pH environments due to river run oﬀ [doi: 10.1016/j.scitotenv.2020.142838.]. The low pH 7.2-7.6 had been observed previously in this area [doi: 10.1007/s11356-022-21122-z.], and pH 7.4 representing coastal acidification had been used for other estuarine species, such as oyster, in previous studies [doi: 10.1111/mec.16751.].

- Furthermore, three pH treatment-level replications were used for the mussels in this study. For each pH treatment-level replication, the mussels with intact-shell and damaged-shell were mixed and raised in the same tank. Six tanks were prepared for the mussels and 30 mussel individuals, including 15 intact-shell and 15 damaged-shell mussels were raised in each tank. A total of 180 mussels were used for our experiment, and 45 individuals for each group.

**Results:**

- The standard curve of FAA analysis used for qualitative and quantitative analysis of free amino acids and other nitrogenous compounds should be provided as supplementary file.

- Also in the section of FAA analysis, the authors used “μg/g wet tissue” as the unit of free amino acids content in Table 1, and “μg/g dry tissue” in other place. Which one is correct?

**Response**: thanks for reviewer’s suggestions.

- The standard curve of FAA analysis was added in the revised manuscript as S5 Fig according to the reviewer’s suggestion.

- We used μg/g of dry tissue as unit of the FAA analysis result, and the “μg/g wet tissue” is a mistake and we corrected accordingly.

**Discussion:**

- The authors should clearly specify which comparisons were performed and base the interpretation of results on those specific comparisons. This will help avoid any misinterpretation of the findings. For example, in Line 415, “The SDMs in various comparisons…”, and Line 445, “The up-regulation of GSSG under the acute acidification…”.

In addition, the following articles might be decent for authors to further strengthen their discussion.

https://doi.org/10.1021/acs.est.1c06735

https://doi.org/10.1016/j.scitotenv.2022.156442

https://doi.org/10.1016/j.chemosphere.2019.125415

**Response**: thanks for reviewer’s suggestions. We revised some ambiguous descriptions in Discussion, and one of the suggested references was added.

**Other minor errors:**

Some typo errors should be corrected. Such as:

- In the section of Enzymatic assay, “described in the section 2.1”, where is the section 2.1?

- In Line 386, “no mark changes” should be “no marked changes”;

- In Discussion, paragraph six, “overserved” should be “observed”; in the next paragraph, “as one of negative effect for…” should be “as one of negative effects on…”, etc.

**Response**: thanks for reviewer’s suggestions. The errors were corrected and the English of this manuscript was re-edited by Editage (www.editage.com).

**Journal requirements:**

**1. Please ensure that your manuscript meets PLOS ONE's style requirements, including those for file naming. The PLOS ONE style templates can be found at**

https://journals.plos.org/plosone/s/file?id=wjVg/PLOSOne_formatting_sample_main_body.pdf and https://journals.plos.org/plosone/s/file?id=ba62/PLOSOne_formatting_sample_title_authors_affiliations.pdf

**Response**: thanks for Editor’s suggestions. We checked and revised our manuscript to meet the PLOS ONE's style requirements

**2. We suggest you thoroughly copyedit your manuscript for language usage, spelling, and grammar. If you do not know anyone who can help you do this, you may wish to consider employing a professional scientific editing service.**

Whilst you may use any professional scientific editing service of your choice, PLOS has partnered with both American Journal Experts (AJE) and Editage to provide discounted services to PLOS authors. Both organizations have experience helping authors meet PLOS guidelines and can provide language editing, translation, manuscript formatting, and figure formatting to ensure your manuscript meets our submission guidelines. To take advantage of our partnership with AJE, visit the AJE website (http://learn.aje.com/plos/) for a 15% discount off AJE services. To take advantage of our partnership with Editage, visit the Editage website (www.editage.com) and enter referral code PLOSEDIT for a 15% discount off Editage services.  If the PLOS editorial team finds any language issues in text that either AJE or Editage has edited, the service provider will re-edit the text for free.

Upon resubmission, please provide the following:

The name of the colleague or the details of the professional service that edited your manuscript

A copy of your manuscript showing your changes by either highlighting them or using track changes (uploaded as a *supporting information* file)

A clean copy of the edited manuscript (uploaded as the new *manuscript* file)

**Response**: thanks for Editor’s suggestions. The manuscript was polished by Editage (www.editage.com), and the language usage, spelling, and grammar were revised thoroughly. The Editing Certificate was attached below.

**3. Thank you for stating the following financial disclosure:**

“This study was supported by the National Natural Science Foundation of China (Grant Nos. 32271580, 42020104009, and 32200083). Zhejiang Provincial Natural Science Foundation (Grant Nos. LQ23D060002 and LTGS23C010001).”

Please state what role the funders took in the study.  If the funders had no role, please state: "The funders had no role in study design, data collection and analysis, decision to publish, or preparation of the manuscript."

If this statement is not correct you must amend it as needed.

Please include this amended Role of Funder statement in your cover letter; we will change the online submission form on your behalf.

**Response**: the role the funders took in this study was stated in the section of Founding,

**4. Thank you for stating the following in the Acknowledgments Section of your manuscript:**

“ This research was supported by the National Natural Science Foundation of China (Grant Nos. 32271580, 42020104009, and 32200083). Zhejiang Provincial Natural Science Foundation (Grant Nos. LQ23D060002 and LTGS23C010001).”

We note that you have provided funding information that is currently declared in your Funding Statement. However, funding information should not appear in the Acknowledgments section or other areas of your manuscript. We will only publish funding information present in the Funding Statement section of the online submission form.

Please remove any funding-related text from the manuscript and let us know how you would like to update your Funding Statement. Currently, your Funding Statement reads as follows:

“This study was supported by the National Natural Science Foundation of China (Grant Nos. 32271580, 42020104009, and 32200083). Zhejiang Provincial Natural Science Foundation (Grant Nos. LQ23D060002 and LTGS23C010001).”

Please include your amended statements within your cover letter; we will change the online submission form on your behalf.

**Response**: the founding information in Acknowledgments Section was deleted.

5. We note that you have stated that you will provide repository information for your data at acceptance. Should your manuscript be accepted for publication, we will hold it until you provide the relevant accession numbers or DOIs necessary to access your data. If you wish to make changes to your Data Availability statement, please describe these changes in your cover letter and we will update your Data Availability statement to reflect the information you provide.

**Response**: thanks for Editor’s suggestion. We accept that we will provide the relevant accession numbers or DOIs necessary to access our data if our manuscript be accepted for publication.

6. We are unable to open your Supporting Information file [Supporting information.rar]. Please kindly revise as necessary and re-upload.

**Response**: we are sorry for that. We previously compressed our Supporting Information file using RAR format, and we will provide the Supporting Information files using ZIP format.
